# An immunocompetent patient presenting with severe septic arthritis due to *Ralstonia pickettii* identified by molecular-based assays: a case report

**DOI:** 10.4076/1757-1626-2-8125

**Published:** 2009-07-16

**Authors:** Konstantinos P Makaritsis, Charalambos Neocleous, Nikolaos Gatselis, Efthimia Petinaki, George N Dalekos

**Affiliations:** 1 Department of Medicine, Medical School, University of Thessaly41110 LarissaGreece; 2 Department of Microbiology, Medical School, University of Thessaly41110 LarissaGreece; 3 Institute of Biomedical Research and Technology, Centre for Research and Technology-Thessaly (CE.RE.TE.TH)LarissaGreece

## Abstract

**Introduction:**

*Ralstonia pickettii* is an infrequent pathogen of invasive infections in healthy individuals. The microorganism is supposed to be of relatively low virulence, but can cause infections, mainly of the respiratory tract, in immunocompromised and cystic fibrosis patients. *Ralstonia pickettii* has also been associated with hospital outbreaks related to contamination of products used for medical care and laboratory diagnosis.

**Case presentation:**

We report here a case of septic arthritis due to *Ralstonia pickettii* in a female diabetic patient. The microorganism was identified from the synovial fluid by molecular-based methods, while the conventional synovial and blood cultures proved to be negative. The patient was treated by intravenous ceftazidime with complete remission of her symptoms; she was discharged 3 weeks after admission in a very good health. At follow-up examination 3 weeks later, she was still in good health condition without any sign of arthritis of the right knee and afebrile.

**Conclusion:**

In culture negative serious bacterial infections, as septic arthritis, the use of molecular-based techniques might be of outmost importance as additional and rapid diagnostic tools for the identification of the causative agent allowing a prompt and appropriate antimicrobial therapy and a favourable outcome.

## Introduction

*Ralstonia pickettii*, formerly known as *Pseudomonas (Burkholderia) pickettii*, is a non-fermenting gram-negative bacillus that is found in water, soil, plants, fruits and vegetables [1]. Infections due to *Ralstonia pickettii* are very rare in healthy individuals, since the microorganism is supposed to be of relatively low virulence and is often associated with pseudobacteremia or asymptomatic colonization of the respiratory tract as, for instance, in cystic fibrosis patients [2]. However, *Ralstonia pickettii* hospital outbreaks have been reported and associated with extrinsic contamination of disinfectants, saline solution, sterile water and other solutions used for patient care [3,4]. Herein, we describe a case of septic arthritis caused by *Ralstonia pickettii* in a female diabetic patient. Of interest, the microorganism was identified rapidly and directly from the clinical sample by molecular techniques, while the conventional culture methods proved to be negative.

## Case presentation

An 83-year-old female Greek patient of Caucasian origin patient was admitted to the Department of Medicine, Medical School University of Thessaly, Larissa, Greece because of fever and severe arthritis of the right knee. Her past medical history was positive of arterial hypertension, adult-onset diabetes mellitus and an episode of ischemic stroke. She was on treatment with amlodipine, moxonidine, valsartan, clopidogrel, atorvastatin and sc insulin. On admission the patient was febrile (38°C) and was complaining for pain at the right knee for the last 15 days. Physical examination revealed warmth, redness and swelling in the area of the right knee and a harsh systolic murmur (2-3/6) that was heard along the left sternal border radiating to the neck. The remaining physical examination was unremarkable. Blood pressure was 144/88 mmHg, heart rate was 98 beats per min and her respiratory rate was 16 per min. White blood cell count (10.5 × 10^9^/L), erythrocyte sedimentation rate (80 mm/1h), blood glucose (170 mg/dL) and C-reactive protein (127 mg/dL; upper normal limit: 10 mg/dL) were elevated. The remaining haematological and biochemical parameters were within normal limits. Synovial fluid aspiration was performed by a sterile syringe and the sample was then immediately transported to the clinical microbiology laboratory. The fluid was grossly purulent with 35,000/μL leukocytes (92% polymorphonuclear cells), although no microorganisms were seen on Gram staining. Light microscopy of the aspirated material did not show the presence of any crystals. Empirical intravenous antibiotic treatment was started with 2 g of ceftriaxone b.i.d. and 500 mg of daptomycin q.d. at this time for septic arthritis. A trans-thoracic echocardiogram was unrevealing.

An aliquot of synovial fluid was inoculated directly onto 5% sheep blood agar and chocolate agar, which were incubated at 35°C with 5 to 7% CO_2_ for 7 days; in parallel, 3 ml of the synovial fluid were inoculated into a BACTEC Peds Plus/F bottle, which was loaded into the BACTEC 9240 instrument and incubated for 7 days. In addition, total DNA and RNA were extracted from a volume of 2 ml of synovial fluid. Then, 5 μl of DNA was used as template for the detection of bacterial DNA, using the *16 S rRNA* PCR assay as we have described previously [5,6]. After amplification, the PCR product was purified and sequenced, and the obtained sequences (approximately 460 bp) were compared with the *16S rRNA* sequences available both in the Ribosomal Database Project and the Genbank and EMBL, obtained from the National Center for Biotechnology Information Database by the advanced BLAST search. The viability of the microorganism was assessed by reverse-transcription of the extracted RNA (Invitrogen), followed by PCR using the primers described previously [5,6]. The results showed the presence of *Ralstonia pickettii*, while the viability test confirmed that the microorganism was viable. The results were available within three days from the date of specimen collection. At this point the treatment was changed to intravenous administration of 2 g t.i.d. of ceftazidime as the patient had not responded to the previous antibiotic regimen. Intravenous antibiotic treatment with ceftazidime was continued for 3 weeks. Laboratory inflammatory indices returned back to normal gradually, the fever disappeared and no repeated drainage was required as the arthritis resolved completely. The patient was discharged 3 weeks after admission in a very good health. At follow-up examination 3 weeks later, she was still in good health condition without any sign of arthritis of the right knee and afebrile. Of note, the conventional synovial fluid cultures and consecutive blood cultures failed to detect the microorganism; after a prolonged incubation of fifteen days no growth of microorganisms was observed.

## Conclusion

A few number of reports in the English literature indicate that *Ralstonia pickettii* may be responsible for more invasive and severe infections than was previously thought. Indeed, reports on bacteremia and septicemia caused by *Ralstonia pickettii* have already been described [7,8]. Occasionally, *Ralstonia pickettii* has been identified as the causative agent in infectious endocarditis [9], meningitis [10], seminal infection [11], osteomyelitis or spondylodiskitis [12] and septic arthritis [13]. Immunocompromised patients and those with cystic fibrosis are also at increased risk of acquiring infection with *Ralstonia pickettii* [14,15]. Other underlying conditions that have been implicated in *Ralstonia pickettii* invasive infections include alcoholic and hepatitis C related cirrhosis, chronic renal failure and diabetes mellitus [16].

Although *Ralstonia pickettii* is not a fastidious microorganism, in our hands the conventional cultures of synovial fluid and blood samples were negative. The latter could be attributed to the fact that this microorganism in general, is characterized by a relatively low inoculum. In addition, it is well known that conventional cultures may lack the sensitivity and specificity to establish an aetiologically definite diagnosis in cases of septic arthritis. It seems that at least in some cases, broad-range *16 S rRNA* PCR assay followed by sequencing might be of outmost importance in an attempt to successfully identify microorganisms involved in such infections like septic arthritis and in particular, when patients have previously received antibiotics or in the presence of slow-growing or intracellular microorganisms [5,17]. In this context, our findings further emphasize the potential usefulness of molecular-based methods as additional and rapid diagnostic tools for the identification of the causative agent of serious bacterial infections. Furthermore, the use of a viability test in case of a positive result by the *16 S rRNA* PCR assay gives an additional advantage of the molecular-based assays as with this test we are capable to assess whether the causative microorganism is alive or not. However, the use of these assays should be rather restricted mainly in culture-negative cases and when serious infections are suspected on the basis of clinical signs and symptoms [5].

## Abbreviations

bp: Base Pairs
CO_2_: Carbon dioxide
DNA: Deoxyribose Nucleic Acid
PCR: Polymerase Chain Reaction
RNA: Ribose Nucleic Acid
rRNA: Ribosomal RNA
sc: Subcutaneous
